# Who's afraid of *Homo sapiens*?

**DOI:** 10.1186/1747-5333-1-17

**Published:** 2006-11-29

**Authors:** Todd M Preuss

**Affiliations:** 1 Division of Neuroscience, and Center for Behavioral Neuroscience, Yerkes National Primate Research Center, Emory University, 954 Gatewood Road, Atlanta, GA 30329, USA

## Abstract

Understanding how humans differ from other animals, as well as how we are like them, requires comparative investigations. For the purpose of documenting the distinctive features of humans, the most informative research involves comparing humans to our closest relatives–the chimpanzees and other great apes. Psychology and anthropology have maintained a tradition of empirical comparative research on human specializations of cognition. The neurosciences, by contrast, have been dominated by the model-animal research paradigm, which presupposes the commonality of "basic" features of brain organization across species and discourages serious treatment of species differences. As a result, the neurosciences have made little progress in understanding human brain specializations. Recent developments in neuroimaging, genomics, and other non-invasive techniques make it possible to directly compare humans and nonhuman species at levels of organization that were previously inaccessible, offering the hope of gaining a better understanding of the species-specific features of the human brain. This hope will be dashed, however, if chimpanzees and other great ape species become unavailable for even non-invasive research.

## Introduction

What a pleasure it is to read Karl Pribram's 1970 James Arthur Lecture on human specializations of the brain and cognition [1]. It takes us back to a time of bold scientific conjectures about human nature, an era that saw the publication of Konrad Lorenz's *On Aggression *(1966; first German edition, 1963) [2], B.F. Skinner's *Beyond Freedom and Dignity *(1971) [3], and E.O. Wilson's *Sociobiology *(1975) [4], to take a few examples, all books that intrigued me (and sometimes infuriated me) as a student. Pribram displays an impressively broad knowledge of psychology and neuroscience, achieving a synthesis that few scientists at the time would have been capable of and that even fewer scientists could credibly attempt today. The work is also impressive because the author is reasonably specific about what he considers to be the distinctive characteristics of human beings. In the realm of cognition, he emphasizes meaning, and in particular the ability of humans to construct propositions. He doesn't quite claim that language is a signature specialization of human cognition, but this is implicit in his argument. He considers, for example, that chimpanzees can use signs, but is not impressed with their syntactic capacity. In the realm of neuroscience, Pribram is less specific, but he does suggest that the distinctiveness of the human brain involves not the organization of cortico-cortical connections, as one might suppose, but rather the organization of cortical connections with subcortical motor structures, consistent with his view that meaning is grounded in intention and action.

How do Pribram's conjectures hold up in the light of modern research? It must be acknowledged that Pribram's lecture did not spark a new set of investigations into human cognitive or neurological specializations. In the 1970s, comparative human-ape research was dominated by the ape-language project, which involved teaching apes (mainly chimpanzees) to communicate using systems of manual gesture, such as American Sign Language, or with physical tokens. Popular culture today takes it as given that apes share with humans the capacity for language. Students of language, however, have for the most part drawn the opposite lesson from the ape-language project, concluding that apes demonstrate little productive language capacity, even after intensive training, and that language is a human specialization (e.g., [5-12]). Recent years have brought new ideas about human cognitive specializations. For example, Povinelli [13-15] has proposed that unlike humans, chimpanzees do not form explicit representations of abstract, unobservable variables, such as mental states (in the case of behavioral causation) or mass force (in the case of physical interactions). These proposals have prompted much debate in the comparative cognition community, along with new experiments (to get the flavor of the debate, see Tomasello et al. [16] and accompanying commentaries). It is noteworthy that Povinelli's "reinterpretation" hypothesis [14,17,18], which holds that humans create new causal interpretations of ancient behaviors, recalls Pribram's claim that humans create and represent meaning through propositions. Additionally, anthropologists have revived the classical idea that culture is a human adaptation, and have begun to characterize cognitive specializations involved in the acquisition and transmission of cultural knowledge (e.g., [19-21]).

## What are the evolutionary specializations of the human brain?

While a thin but vital thread of research on human cognitive specializations runs through psychology and anthropology from the 1970s to the present, in the neurosciences the thread has been drawn so fine as to be nearly invisible. Consider Pribram's list of human brain specializations: brain enlargement, hemispheric dominance and specialization, and "somewhat more generalized" cytoarchitecture. Of these, brain enlargement (encephalization) remains well accepted, although the nature of this enlargement–was there a general enlargement or a more specific enlargement of association cortex?–is controversial [22-26]. Whether humans show greater hemispheric specialization than do apes and other nonhuman primates is also controversial: there is some evidence that our closest relatives, the chimpanzees and other apes, possess homologues of Wernicke's and Broca's areas, and that these are lateralized in ways that are at least qualitatively similar to humans [27-30]. There are, however, those who maintain that humans show extreme hemispheric asymmetry and functional laterality, and that these are among the defining features of humans [31-33].

A few human specializations have been identified at finer levels of structural organization. For example, the histological organization of human primary visual cortex displays some striking differences with apes and monkeys [34]. Humans have several populations of morphologically and biochemically distinctive pyramidal cells in anterior cortex [35-37]. The spacing and cell density of minicolumns differ in Wernicke's area of the left and right hemispheres in humans, but not in chimpanzees or monkeys [38]. With the extension of functional imaging techniques to nonhuman primates has come evidence that the functions of homologous visual areas differ to some extent between humans and macaque monkeys (the animals most commonly used as models of the human visual system), and also raise the possibility that humans have several higher-order visual areas not present in monkeys [39-41]. Unfortunately, there have not yet been functional imaging studies of ape visual cortex, so it is not possible to say whether these differences represent true human specializations or specializations of the larger group (the Hominoidea) to which both humans and apes belong; the possibility that differences represent specializations of macaques also needs to be considered. One can point to additional differences between humans and various "model" species, such as human-macaque or human-mouse differences–some of them quite remarkable [42]–but as interesting and important as these differences undoubtedly are, we cannot safely  conclude that these are human specializations without studies that directly compare humans to apes, as well as to other species.

What slim pickings! It seems extraordinary that neuroscience has so little to offer on a matter so fundamental as what is it about our brains that makes us human. No less disturbing is the understanding that if we don't have the information to say much about how our brains *differ *from those of other animals, we probably know less than we suppose about how humans *resemble *other animals, also.

## Why do we know so little about human brain specializations?

How is it that after more than 125 years of experimental neuroscience we know so little about how the human brain differs from that of other species? Consider the kind of research required to understand the place of humans among animals: one would need studies that examine human brain structure and function in detail, and that compare humans to other species using comparable techniques. The most informative comparisons, if one's goal is to understand human specializations, are with the great apes, and especially with chimpanzees (*Pan troglodytes*) and bonobos ("pygmy" chimpanzees; *Pan paniscus*), as these are the animals most closely related to us. If one's main goal is to understand what humans share with other animals, comparative studies would still be essential, although in this case one would need to study a broad range of species, of varying degrees of relationship to humans.

We do not have these kinds of studies. The obvious reason for this is it has been difficult to study the human brain directly in much detail. Historically, the most valuable investigative techniques in the neurosciences have required invasive and terminal techniques, or other manipulations (such as genetic manipulations) that we regard as unethical in humans. So, for the most part, neuroscientists study not real humans but surrogate humans, nonhuman species that we believe are similar enough to humans to be informative about the human condition, but not so similar as to be ethically problematic (or at least not prohibitively so). This approach–the model-animal paradigm–has been widely employed in the experimental biomedical sciences, particularly, in preference to the broader, comparative approach that has flourished in some other biological disciplines.

The adoption of the model-animal paradigm brought about important changes in the way scientists understand the role of animals in research [43-46]. In the early part of the 20^th ^century, before the establishment of currently favored model-animal species such as rats, mice, and rhesus monkeys, experimental biologists sought to identify features of biological organization that are shared by a wide variety of species. To identify these features, it was felt necessary to actually compare a wide variety of species. Attitudes changed as the favored model animals became entrenched as research resources: increasingly it was assumed that the experimentally identified characteristics of these species have broad generality and the imperative to empirically demonstrate generality faded. As historians of science have observed, the establishment of breeding colonies of rodents transformed these animals from subjects of research into something more like standardized chemical reagents [43,44,47]. From this perspective, cross-species variation in biological organization is a problem, a potential threat to the standing of one's particular model animal within the larger research community. Species differences, if they are acknowledged at all, tend to be soft-peddled and relegated to the status of noise: it's the commonalities that matter. In this context, the word "basic" has come to mean "common" or "widely shared." As a result, "basic" keeps close company with "same," so that one often hears the expression "the same basic X," where X can be structure, function, organization, or any other attribute of organisms.

This analysis does not imply that the use of nonhuman species as research models of human biological systems or specific diseases is misguided; after all, humans *do *share many features in common with other animals. What is problematic is the mindset fostered by the model-animal paradigm: viewing the biological world primarily through this lens has serious and negative consequences for the scientific enterprise. For one thing, because the model-animal paradigm discourages systematic, rigorous treatment of the similarities and differences across species, we remain in the position *of assuming *generality, rather than *demonstrating *it empirically. This is a particular problem for the neurosciences–mammalian cerebral cortex, for example, has proven to be far more variable across species than believed a decade or two ago, fundamentally compromising the idea that there is a "basic uniformity" of cortical design [48-51]. In addition, by failing to take differences seriously, we have largely ignored the correlated variations between biological organization and function produced by evolution, and in so doing have ignored a very rich source of information about structure-function relationships (for examples of how such variations can be exploited, see [52,53]). Finally, the model-animal paradigm has little room for human specializations, for if we consider to be "basic" only those characteristics of biological organization that are shared among species, then the features that distinguish human brains from those of other species don't count as "basic." These would include some of the most interesting and important things we would like to know about human beings, such as what features of our brains support our distinctive cognitive capacities and what features render humans (alone among primates) vulnerable to Alzheimer's disease [54,55].

## Making room for humans: the critical need for human-chimpanzee comparisons

We can begin to redress some of the deficiencies in our scientific knowledge stemming from the primacy of the model-animal paradigm, with its very indirect approach to understanding human nature, by investing more in comparative studies. The time is especially propitious for studies that address human specializations through direct comparisons of humans and closely related species, because new techniques are available that make it possible to directly study the human brain in great detail, and in ethically acceptable ways, and we can apply these same techniques to the study of our closest relatives.

As the spatial resolution of neuroimaging techniques has improved, it has become practical to use them to compare the structure and function of human brains to those of other primates. While comparisons with macaque monkeys–the favored model nonhuman primates–have been emphasized [56-59], these approaches can be applied to the study of chimpanzees and other ape species as well [60,61], opening the door to new explorations of human-specific brain organization. In addition, histological studies of humans, using tissue obtained postmortem, have undergone something of a rebirth, driven in part by the need to provide a better understanding of the regional organization of human cortex for the correct interpretation of functional imaging results than can be obtained from the hundred-year-old cortical map of Brodmann [62]. It is clear that one can obtain reliable and informative results with postmortem tissue derived not only from humans, as well as from apes and other nonhuman primates (e.g., [34-36,63,64]).

The growth of knowledge about the genomic organization of humans and nonhuman primates, including chimpanzees, creates additional opportunities for understanding human-specific brain organization. A number of studies have identified gene-expression or gene-sequence differences between humans, chimpanzees, and other nonhuman primates (reviewed in [65-67]). Large-scale genomic changes in human evolution have also been documented, involving duplications and rearrangements of DNA, which in some cases have even resulted in the creation of novel, human-specific genes (e.g., [68-70]).

Comparative genomics research on human-ape differences has generated enormous interest in the popular media, which is not surprising given the strong interest of the public in understanding what makes us human. One important point that is not widely appreciated, however, is that the identification of genetic differences that distinguish humans from other animals is ultimately of little value if we cannot connect the genetic differences to phenotypic differences [71,72]. At the present time, the wealth of information about human-chimpanzee genetic differences stands in stark contrast to the poverty of our understanding of human-chimpanzee differences in brain organization. My colleagues and I have suggested that we can use the information from comparative genetic studies as clues to identify previously unknown phenotypic specializations of the human brain, for example, by following the trail from genes to mRNA and protein expression in tissue [66,73]. While we have focused on comparative histological studies, genomics-driven "phenotype discovery" could employ any of the growing array of non-invasive, non-terminal techniques (biochemical, proteomic, imaging, behavioral) we have for comparing humans to other animals.

## Will there be chimpanzees to study?

The advent of technologies like genomics and neuroimaging gives me hope that neuroscience can adopt a more direct approach to the study of human nature and once again deal with fundamental questions of the kind addressed in Karl Pribram's lecture. My optimism is tempered, however, by the knowledge that we might soon lose a resource vital for this pursuit. Understanding what makes our brain distinctively human requires comparing humans to chimpanzees, our closest relatives. In a world where the value of animals in research is measured mainly in terms of their utility as "models," chimpanzees don't fit in very well, as they are more expensive and difficult to maintain than, say, rodents, and as with humans, their use in invasive research is restricted. So, despite the obvious value of comparative studies of chimpanzees and humans for understanding the human brain (including its distinctive vulnerability to neurodegenerative disease), and despite the availability of powerful new techniques for comparing human and chimpanzee brains, it may soon become impossible to pursue this essential research. In 1997, faced with a larger population of captive chimpanzees than could reasonably be supported, NIH imposed a moratorium on breeding chimpanzees. Consequently, the population is not being replaced as animals die of old age and the number of chimpanzees available for the kind of non-invasive research described above–the kind of research we conduct with humans–is rapidly shrinking. Given the highly endangered status of chimpanzees, which seem destined for extinction in the wild within a few decades, it is unlikely that such a resource, once lost, would be reconstituted. In 2007, NIH will decide whether or not to lift the moratorium. As someone committed to understanding the structure, function, and diseases of the human nervous system, I hope the moratorium will be ended. It seems prudent, now that the population has been reduced, to allow enough breeding to maintain a viable population. To be sure, it would be simpler to continue the current policy, avoiding the political heat that would result from choosing to maintain the chimpanzee population, and instead devote our limited resources to supporting mice and a few other favored model-animal species. But, after all, the mouse we will always have with us, whereas chimpanzees will be with us for only a short time longer, if we continue on our current course. If we fail to preserve the means to understand what makes us human, in health and in disease, future generations will surely ask: What *were *they thinking?

## Competing interests

The author(s) declare that they have no competing interests.

## Authors' contributions

TMP is the sole author of this paper.
